# The Opium Habit

**Published:** 1883-07-20

**Authors:** W. D. Wilhite


### THE OPIUM HABIT.

*A Narrative of Personal Experience,*

BY W. D. WILHITE, M. D.

While I regard opium and its salts as at the very head of the list of remedies in the practice of medicine, I at the same time regard it very injurious if the use of it is continued for too long a time.

The opium habit, when once contracted, is more injurious and harder to get rid of than the profession generally realize. I must confess I was very much astonished in talking with physicians to find that they generally knew so little of the magnitude and power of the habit of opium-eating. The great number of opium-eaters is surprising to any one who has not investigated the subject.

I have chosen this subject simply because it is one that deeply concerns the human family, there being so many addicted to the habit. I have seen nothing written on the subject, either in books or medical journals. Our authors, so far as I have examined, are perfectly dumb on the habit and treatment of opium-eating.

I was led to investigate this subject under the peculiar circumstances of being a slave to the habit myself. In the year 1870 I had a severe attack of rheumatism. Was confined to my room four months. I suffered intensely, and to relieve that suffering I used morphia, the great panacea for pain. I thus contracted, unconsciously, the abominable habit.

When I got able to leave my room I thought I was cured. I found I could have no rest when I left off taking the morphia. In the course of time my rheumatism developed into neuralgia, or what I thought was neuralgia, but am satisfied now it was the effect of morphia. So, acting on my judgment at the time, I continued the morphia in larger doses, which would lull me some, but not relieve the pain. I never suffered myself to use morphia to that inordinate extent that I have known some to use it. Therefore it did not supply the demands of the system. I had to resort to something else. I took, in connection with the morphia, Gross' neuralgia pills. I continued in this condition for some time, getting no better, in fact, worse. I concluded to try Brown-Sequard's neuralgia pills with morphia. All the while my system demanding more of the opium. Not willing to increase the dose, I thus kept myself in that condition, suffering all the while, as I thought, from neuralgia. Sometimes I would call it periostitis, so painful would be the part affected, especially on pressure.

I knew the morphia was injuring me. My secretions were all checked, bowels constipated, no appetite, at times my memory was defective, my mind vascillating, no decision of character, in fact, I had no energy. In a semi-comatose condition, only when I was under the exhilerating effect of the morphia, which would only last two or three hours, when the effect would die out partly, leaving me in great pain, which I would bear until my regular time for taking a dose, which was morning and night. I would watch the time with eager anxiety for the hour to come, so distressing was my condition. A physician who makes treating the opium habit a specialty, and has treated more than a thousand patients, told me I had fought it more manfully than any patient he ever saw.

I lost the power of my muscles, so much so I could not put my foot into my stirrup and mount the smallest pony. I had to drag myself into my buggy with my hands, having so little strength in my legs. My muscular system seemed to be flabby—want of tone and power; my heart would flutter and palpitate upon the least exertion or excitement, I think for the want of tone of the walls of the heart, as I do not have those symptoms now.

I have now told you some of the effects of the opium habit on myself. I will now try to tell what I call the indescribable part, in the language of a medical brother, I will call it the "indescribable wretchedness" of feeling produced by leaving off the morphia. I will here say that no one who has thoroughly contracted the habit can be cured if he at the same time attempts to carry on any business. He must leave off everything and give himself entirely to treatment. I made several efforts to leave off suddenly, although I had not been as great a slave to morphia as a patient I once saw who took thirty grains night and morning, yet I found it impossible to leave off suddenly, although I had never taken over two grains at a dose. I well remember, on one occasion, I resolved to quit off at once. I knew the terrible scenes and suffering I would have to undergo, so I fortified myself with a pint of the best whisky. The hour came to take the accustomed dose, and with it the indescribable wretchedness alluded to. But I began on the whisky as an antidote. I drank the pint in less than three hours without any effect. I walked the floor all night long. I was like a wild man. My limbs felt as if they would weigh a ton. My wife, knowing the trouble, advised me to take a small dose of morphia. I did so. It relieved me. Such sweet peace and repose I never shall forget. I then tried the gradual reduction plan, but trying to keep up my business and practice at the same time, I failed. The fact was, I did not realize the magnitude and power of the enemy I had to fight. Failing in all my efforts to get rid of the pernicious habit, I resolved to leave home and business and put myself under treatment at the St. Louis Sanitarium. I remained there some time. The treatment was a gradual withdrawal of the opium, quietude, and good nourishing diet. I will here say, that the only remedy for the opium habit is opium. One great trouble with me in trying to cure myself, I tried to reduce too rapidly. It has to be slow to give the system time to recuperate. As the system rallies, in the same proportion can the opium be reduced. In my case the opium was withdrawn too rapidly at first, my system became prostrated to some extent so that I had to reduce the opiate as I could bear it.

In about three months I was cured, or could go without the opium. The last fourth of a grain was more trouble to get rid of than all the rest. No one that has had no experience in the opium habit can conceive of the magnitude and power of its effects.

The cause of the failure of treating opium-eaters after the old plan is plain to my mind. The absurd idea of confining a patient in a cell and withholding the opium to substitute someting else is perfectly barbarous. This plan of treatment, I am sorry to say, has been adopted by some hospitals. The result was, the patient became crazed, had to be confined in a strait-jacket, relaxation, prostration and diarrhœa set in and a horrible death was the termination.

Gentlemen, in conclusion, I will say, the treatment of the opium habit is that of *similia similibus curantur*.—*St. Louis Clin. Rec.*

